# 182. Back to The Future: Increasing Penicillin Susceptibility among Methicillin-Susceptible *Staphylococcus aureus* Osteoarticular Infections in Children

**DOI:** 10.1093/ofid/ofab466.182

**Published:** 2021-12-04

**Authors:** Jonathon C McNeil, Lauren Sommer, Jesus G Vallejo, Mary G Boyle, Kristina G Hulten, Sheldon L Kaplan, Stephanie Fritz

**Affiliations:** 1 Baylor College of Medicine, Houston, TX; 2 Washington University, St. Louis, MO

## Abstract

**Background:**

Starting in the late 1940s-1950s *Staphylococcus aureus* isolates gained resistance to penicillin largely through the acquisition of β-lactamases. In recent years, some centers have described an increase in the proportion of methicillin susceptible *S. aureus* (MSSA) which are also susceptible to penicillin (PSSA). There are little data on the prevalence or clinical significance of PSSA in children. Acute hematogenous osteoarticular infections (AHOAIs, including osteomyelitis and septic arthritis) are the most common manifestation of invasive *S. aureus* disease in children. We investigated the prevalence of penicillin susceptibility among MSSA AHOAI isolates at two children’s hospitals.

**Methods:**

MSSA AHOAI isolates were obtained through surveillance studies at Texas Children’s (TCH) and St. Louis Children’s Hospitals (SLCH) from 1/2011- 12/2019. All isolates underwent PCR for *blaZ* β-lactamase, PVL genes and *agr* group. All *blaZ* negative isolates then underwent penicillin susceptibility testing using macrobroth dilution. Isolates which were *blaZ* negative and had a penicillin MIC ≤ 0.125 μg/ml were regarded as PSSA.

**Results:**

329 unique isolates were available and included in the study. The median patient age was 9.2 years (IQR: 5.1-12.2). Overall, 22 isolates were found to be penicillin susceptible (6.7%). No PSSA isolates were detected prior to 2015 but increased yearly thereafter; by the final study year 20.4% of isolates were PSSA (p=0.001, **Figure 1**). Patients with PSSA isolates were slightly older than those with resistant isolates (median age 11.8 years vs. 9.1 years, p=0.08) and PSSA were more commonly identified at SLCH (12.9% vs. 5.2%, p=0.04). PSSA were similar to penicillin-resistant isolates in terms *agr* group and PVL carriage as well as clinical presentation and outcomes. For PSSA, the MIC_90 _for penicillin (0.06 μg/ml) was much lower than that for other β-lactams (**Figure 2**).

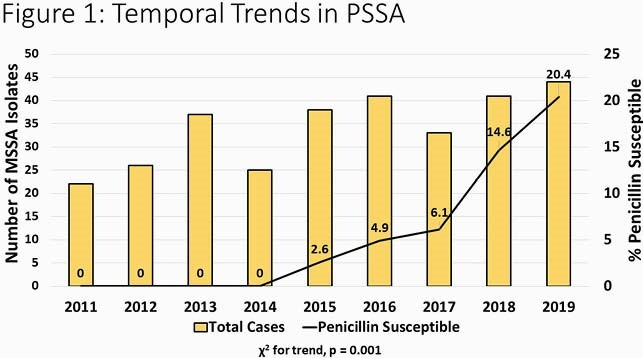

The figure describes the relative frequency of penicillin susceptible S. aureus (PSSA) over time among MSSA osteoarticular infection isolates in children.

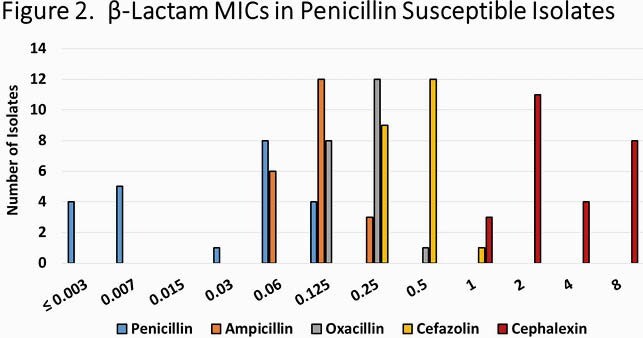

Distribution of MICs to penicillin, ampicillin, cefazolin, cephalexin and oxacillin among PSSA isolates.

**Conclusion:**

PSSA appears to be increasing among AHOAI isolates in US children, although geographic variability does occur. Overall, PSSA isolates are associated with a similar clinical presentation as penicillin-resistant isolates. Penicillin susceptibility testing may serve as an avenue for future stewardship intervention in staphylococcal infections.

**Disclosures:**

**Jonathon C. McNeil, MD**, **Agency for Healthcare Research and Quality** (Research Grant or Support)**Allergan** (Grant/Research Support)**Nabriva** (Grant/Research Support, Other Financial or Material Support, Site PI for a multicenter trial) **Kristina G. Hulten, PhD**, **Pfizer** (Research Grant or Support) **Sheldon L. Kaplan, MD**, **Pfizer** (Research Grant or Support)

